# A Dihydroflavonoid Naringin Extends the Lifespan of *C*. *elegans* and Delays the Progression of Aging-Related Diseases in PD/AD Models via DAF-16

**DOI:** 10.1155/2020/6069354

**Published:** 2020-07-31

**Authors:** Qing Zhu, Yuan Qu, Xiao-Gang Zhou, Jian-Ning Chen, Huai-Rong Luo, Gui-Sheng Wu

**Affiliations:** ^1^Key Laboratory for Aging and Regenerative Medicine, Department of Pharmacology School of Pharmacy, Southwest Medical University, Luzhou, Sichuan 646000, China; ^2^Key Laboratory of Medical Electrophysiology, Ministry of Education, Institute of Cardiovascular Research of Southwest Medical University, Luzhou, Sichuan 646000, China; ^3^Central Nervous System Drug Key Laboratory of Sichuan Province, Luzhou, Sichuan 646000, China

## Abstract

Naringin is a dihydroflavonoid, which is rich in several plant species used for herbal medicine. It has a wide range of biological activities, including antineoplastic, anti-inflammatory, antiphotoaging, and antioxidative activities. So it would be interesting to know if naringin has an effect on aging and aging-related diseases. We examined the effect of naringin on the aging of *Caenorhabditis elegans* (*C*. *elegans*). Our results showed that naringin could extend the lifespan of *C*. *elegans*. Moreover, naringin could also increase the thermal and oxidative stress tolerance, reduce the accumulation of lipofuscin, and delay the progress of aging-related diseases in *C*. *elegans* models of AD and PD. Naringin could not significantly extend the lifespan of long-lived mutants from genes in insulin/IGF-1 signaling (IIS) and nutrient-sensing pathways, such as *daf*-*2*, *akt*-*2*, *akt*-*1*, *eat*-*2*, *sir*-*2*.*1*, and *rsks*-*1*. Naringin treatment prolonged the lifespan of long-lived *glp*-*1* mutants, which have decreased reproductive stem cells. Naringin could not extend the lifespan of a null mutant of the fox-head transcription factor DAF-16. Moreover, naringin could increase the mRNA expression of genes regulated by *daf*-*16* and itself. In conclusion, we show that a natural product naringin could extend the lifespan of *C*. *elegans* and delay the progression of aging-related diseases in *C*. *elegans* models via DAF-16.

## 1. Introduction

Aging is accompanied by constant changes in morphology and gradual decline in function. Aging is a major risk factor for human diseases including cancer, diabetes, cardiovascular diseases, and neurodegenerative diseases [1–3]. Aging could be slowed by getting rid of unhealthy habits, including smoking, bad diet, alcohol consumption, lack of sleep, stress, and sun exposure, and by treating signs of aging with various esthetic methods or food supplements such as antioxidants [4]. Many epidemiological studies have shown that natural bioactive products could reduce the risk of aging-related diseases [5–7]. A number of natural bioactive products, including phosvitin [8], royal jelly-collagen peptide [9], Alaskan berry extracts [10], walnut protein hydrolysate [11], and tea extracts [12], exhibit longevity extension abilities.

**Figure 1:**
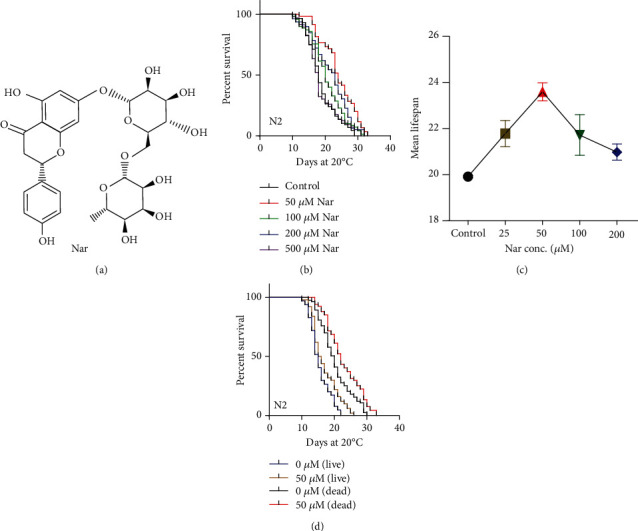
Naringin extended adult lifespan in *C*. *elegans*. (a) Chemical structure of naringin. (b) Survival curves of wild-type (N2) animals raised at 20°C on NGM plates containing 50, 100, 200, and 500 *μ*M of naringin or control plates without naringin. (c) Dose-response analysis of naringin. Wild-type (N2) animals were treated with 25, 50, 100, and 200 *μ*M of naringin at least two independent experiments, plotting the mean changes of lifespan. Error bars represent the standard deviation (SD). (d) Effects of naringin on the lifespan of wild-type (N2) animals raised at 20°C on NGM plates in the absence (0 *μ*M) or presence (50 *μ*M) of naringin with two different feeding procedures including live bacteria (*E*. *coli* OP50) and dead OP50 (65°C 30 min).

Naringin, also known as citrus or isohesperidin, is a kind of dihydroflavonoid (Figure 1(a)), which is a natural pale yellow pigment that exists in the peel and fruit of Citrus grandis, Citrus paradisi, and Citrus (Rutaceae) aurantium [13]. Naringin exhibits multiple biological activities and pharmacological effects, including antitumor [14], antihypercholesterolemic [15], desensitization [16], antiallergy, antiphotoaging [17], cytoprotective [18], anti-inflammatory, heart-protective [7, 13, 19, 20], and neuroprotective activities [21–23]. Naringin could also regulate glucose and lipid metabolism [24] and oxidative stress [25]. Given the various biological activities reported, we are wondering if naringin has an effect on aging and aging-related diseases. We found that naringin could significantly extend the lifespan of *C*. *elegans* and delay aging-related degeneration in body movement and delay the progression of aging-related diseases in models of Alzheimer's and Parkinson's diseases.

## 2. Materials and Methods

### 2.1. Chemicals and Strains of *C*. *elegans*

All strains were from *Caenorhabditis* Genetics Center (CGC) and maintained (unless otherwise stated) at 20°C on nematode growth medium (NGM) agar plates carrying a lawn of *Escherichia coli* OP50 as described previously [26]. The strains used in this study were Bristol N2 (wild-type), DA1116 *eat*-*2*(*ad1116*)*II*, CL4176 *smg*-*1*(*cc546*)*I*, PS3551 *hsf*-*1*(*sy441*)*I*, CF1903 *glp*-*1*(*e2141*)*III*, TJ356 *zIs356* [*Pdaf*-*16*::*daf*-*16*-*gfp*; *rol*-*6*]*IV*, CB4876 *clk*-*1*(*e2519*)*III*, VC204 *akt*-*2*(*ok393*)*X*, CF1553 *muIs84* [*Psod*-*3*::*GFP*], CF1038 *daf*-*16*(*mu86*)*I*, CB1370 *daf*-*2*(*e1370*)*III*, RB759 *akt*-*1*(*ok525*)*V*, VC199 *sir*-*2*.*1*(*ok434*)*IV*, and BZ555 (*P*^dat–1^::gfp). CL4176 *smg*-*1*(*cc546*)*I* was temperature-sensitive [27] and was maintained at 15°C and shifted to 25°C at the L3 stage in lifespan experiments. For CF1903 *glp*-*1*(*e2141*)*III*, L1 larvae were cultured at 25°C until they develop into L4 larvae or young adults, then switched to 20°C for the lifespan test [28]. Naringin was purchased from Sigma and completely dissolved in PBS and then added to the top of the prepared plates. NGM plates containing naringin were equilibrated overnight before use. Synchronized late L4 larvae or young adult worms (wild-type) were transferred to NGM plates containing naringin and maintained at 20°C.

### 2.2. Lifespan Assay

All strains were cultured on fresh NGM plates at least for 2-3 generations without starvation, and lifespan analyses were performed in the same manner at 20°C, unless otherwise stated. Late L4 larvae or young adults were transferred to NGM plates containing inactive OP50 (65°C for 30 min) and 40 *μ*M of 5-fluoro-2′-deoxyuridine (FUDR, Sigma) to prevent progeny growth [29]. The time L4 larvae or young adults were transferred to a NGM plate was defined as test day 0. Live and dead animals were counted each day until all individuals have died. The worms that were not active when gently prodded using a platinum wire were scored as dead [29]. Besides, the worms were transferred to fresh plates every other day. Worms suffering from internal hatch and crawling off the NGM plate were not included in the lifespan counts. The lifespan assays were repeated for at least three independent trials. At least 60 animals were included in each group of lifespan experiment.

### 2.3. Aging-Related Phenotype Analysis

The body movement assay was conducted as previously described [29]. Late L4 larvae or young adults were transferred to NGM plates and treated as described in the lifespan assay. The bending behavior in a coordinated sinusoidal manner was counted.

Lipofuscin accumulation assay was conducted as previously reported [30–32]. Wild-type animals at L4 larvae or young adults were treated with naringin; then, the intestinal autofluorescence of lipofuscin was analyzed on the 2nd and 5th days of adulthood. The intestinal autofluorescence of lipofuscin was captured with a Leica epifluorescence microscope using the GFP filter set (with excitation at 340-380 nm and emission at 435-485 nm) and analyzed by using the image processing software ImageJ. The total number of worms in each group of the aging-related phenotype analysis was at least 20.

### 2.4. Stress Resistance Assays

Wild-type N2 worms were pretreated with naringin, followed by oxidation and heat stress treatment. For the oxidative stress experiment, larvae and early adults of stage L4 were treated with 50 *μ*M of naringin for 7 days, then exposed to paraquat (20 mM) and cultured at 20°C, and their death rate was calculated every day [29]. For the heat stress resistance experiment, stage L4 larvae and young adults were treated with 50 *μ*M of naringin for 7 days. Then, the NGM plates were incubated at 35°C. The death rate was calculated every 2 hours [29]. For the pathogenic stress induced by pseudomonas, stage L4 larvae and young adults were treated with 50 *μ*M of naringin for 7 days. Then, the worms were transferred to a new NGM containing pseudomonas and the death of animals was monitored every day. The dead animals of the stress resistance assays were counted the same way as in the lifespan assay. The experiments were repeated independently at least twice. The number of worms in each group of experiments was at least 60.

### 2.5. Aging-Related Disease Analysis

The CL4176 (*dvIs27* (*myo*-*3/A beta 1*-*42/let UTR*)*+pRF4*(*rol*-*6*(*su1006*)) worms were incubated at 15°C until the L3 stage [27, 33], then transferred to NGM plates containing 50 *μ*M of naringin and incubated at 25°C. Paralyzed nematodes were counted every 2 hours. This experiment was independently repeated for at least twice. The total number of worms in each group of experiment was at least 60.

Transgenic nematode strain NL5901 expresses human *α*-synuclein gene fused with yellow fluorescent protein (YFP) [34]. Worms of NL5901 were treated with 50 *μ*M of naringin for 7 days; then, the aggregation of *α*-synuclein was captured with a Leica epifluorescence microscope and analyzed by using the image processing software ImageJ. The experiments were independently repeated at least three times. The number of worms in each group of experiment was at least 30.

The transgenic strain BZ555 (*P*^dat–1^::gfp) has GFP expressed specifically in dopaminergic neurons, which could be induced to degeneration by 6-OHDA [34, 35]. To induce selective degeneration of DA neurons, the L3 stage larvae of strain BZ555 (*P*^dat–1^::gfp) were transferred to the OP50/s-medium containing 50 *μ*M of 6-OHDA and 10 mM of ascorbic acid, incubated at 20°C for 1 h, and gently shaken every 10 minutes [33]. Then, the worms were washed with an M9 buffer three times and cultured in OP50/NGM plates containing 50 *μ*M of naringin for 72 hours [34]. After that, fluorescent photos of head neurons were taken using a Leica epifluorescence microscope (DFC 7000T) and analyzed by using the image processing software ImageJ. The experiments were repeated independently at least twice. The number of worms in each group of experiment was at least 30.

### 2.6. DAF-16::GFP Location and SOD-3::GFP Assay

The subcellular locations of DAF-16::GFP were determined using the transgenic strain TJ356 *daf*-*16*(*zls356IV*). L4 larvae were transferred to the plates containing 50 *μ*M of naringin and cultured for 48 h at 20°C [29, 32]. The image of DAF-16::GFP signal was captured by using a fluorescence microscope system (Leica, DFC 7000T) and analyzed by using the image processing software ImageJ. The experiments were repeated independently at least twice. The total number of worms in each group of experiment was at least 30.

The *C*. *elegans* transgenic strain CF1553 *muIs84* [*Psod*-*3*::*GFP*] expresses GFP fused with SOD-3. The worms of CF1553 were treated with 50 *μ*M naringin for 7 days for lifespan assays [32]. Then, intensity of fluorescence of worms was observed by using a fluorescence microscope system (Leica, DFC 7000T). All fluorescent photos of at least 30 animals in each group were scored by ImageJ, and the experiment was repeated independently at least twice.

### 2.7. ROS Assay

For intracellular ROS accumulation, age-synchronized N2 worms (L4 stage) were treated with 50 *μ*M of naringin at 20°C for 7 days. The positive control group was treated with 2 mM of hydrogen peroxide (H_2_O_2_) solution while the negative control group was treated with 5 mM of antioxidants *N*-acetylcysteine (NAC) [36]. ROS formation was quantified with H2DCF-DA. After being treated with naringin, worms were collected by washing off the plate with an M9 buffer to a centrifuge tube. OP50 was removed by washing the plate three times. Then, 50 *μ*M of H2DCF-DA was added and the worms were incubated for 1 h in the dark at 20°C. After that, the worms were mounted on a glass slide and paralyzed by the addition of tetramisole hydrochloride, and at least 20 worms were randomly photographed using a fluorescence microscope (Leica, DFC 7000T) using a DAPI filter set (with excitation at 488 nm and emission at 525 nm) [32]. ImageJ software was then used to measure the relative fluorescence intensity of the full body. The experiment was repeated at least twice in independent trials with 20 worms per plot.

### 2.8. Reproduction Assay

Individual worms from synchronized L4 larvae were transferred to NGM plates containing 50 *μ*M naringin. Then, the worms from each plate were transferred into a fresh NGM plate at almost the same time every day. The number of progeny was counted each day [37]. The number of worms in each group was more than 20. The experiments were repeated independently at least twice.

### 2.9. Quantitative RT-PCR Assay

About 3,000 synchronized young adult wild-type worms were transferred to 4 NGM plates containing 50 *μ*M of naringin or control plates and cultured at 20°C for 48 h. The RNA was extracted using RNAiso Plus (Takara) and converted to cDNA using a High-Capacity cDNA Reverse Transcription Kit (Applied Biosystems). The qRT-PCR was performed in Power SYBR Green PCR Master Mix (Applied Biosystems) and run by the QuantStudio 6 Flex system. The relative expression of genes was calculated using the 2^–*ΔΔ*CT^ method and normalized to the expression of gene *cdc*-*42* [29]. All the primers used in this research are listed in Table S12.

### 2.10. Statistical Analyses

Lifespan statistical analyses were performed using the SPSS package. Kaplan-Meier lifespan analysis was performed, and *p* values were calculated using the log-rank test. Other results are expressed as the mean ± SEM, and *p* values were calculated by two-tailed *t*-test. *p* < 0.05 was considered significant.

## 3. Results

### 3.1. Naringin Extends the Lifespan of *C*. *elegans*

To investigate if naringin could extend the lifespan of *C*. *elegans*, the wild-type *C*. *elegans* N2 was treated with naringin from stage L4 larva or early adult till their death. Our results showed that naringin increased the lifespan of *C*. *elegans* in a dose-dependent manner (Figure 1(b), Table S1). The 50 *μ*M of naringin had the greatest effect on longevity, extending adult mean lifespan by up to 23% at 20°C (*p* < 0.001) (Figure 1(b), Table S1). Worms exposed to either higher or lower concentrations of naringin showed a smaller or an insignificant lifespan extension (Figure 1(c), Table S1).

The metabolites produced by live bacteria proliferation could significantly shorten the lifespan of *C*. *elegans* [38]. To test if the effect of naringin on the lifespan extension could be affected by live bacteria, worms were fed with live and heat-killed bacteria when treated with 50 *μ*M of naringin. We observed that the mean lifespan of worms fed by heat-killed (nonproliferating) bacteria *E*. *coli* and live bacteria were both significantly increased under the treatment of naringin (Figure 1(d), Table S1), suggesting naringin did not exert on bacteria to extend the lifespan of *C*. *elegans*. Therefore, the dead bacteria were used throughout the experiments.

### 3.2. Naringin Delayed Aging-Related Decline of Phenotypes

**Figure 2:**
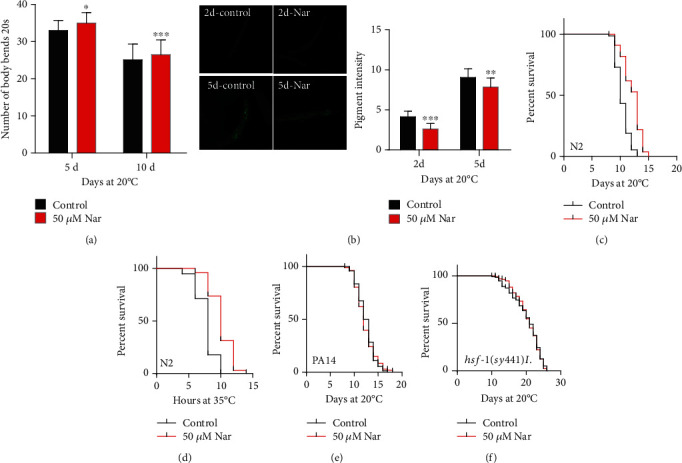
Naringin delayed aging-related decline of phenotypes and improved stress resistance. (a) Aging-related movements of worms with nontreated control plates and 50 *μ*M naringin. The mean body movement speed is found in Table S2 (Supplementary information). (b) The intestinal autofluorescence of lipofuscin was analyzed on the 2nd and 5th days of adulthood. The figures showed the mean lipofuscin aggregated in the intestinal tract at least repeated twice. The statistical details and repeats of these experiments are summarized in Table S3 (Supplementary information). (c) The survival curves of wild-type worms cultured at 20°C in plates with treated 50 *μ*M and nontreated naringin on the 7th day of adulthood; then, the worms were exposed to paraquat (20 mM) and cultured at 20°C; and their death was calculated every day. Statistical details and repeats of these experiments are summarized in Table S4 (Supplementary information). (d) The survival curves of wild-type worms cultured at 35°C in nontreated control plates and plates treated with 50 *μ*M naringin. Statistical details and repeats of these experiments are summarized in Table S5 (Supplementary information). (e) Survival curves of wild-type (N2) animals feeding Pseudomonas aeruginosa raised at 20°C on NGM plates in the absence (0 *μ*M) or presence (50 *μ*M) of naringin. Statistical details and repeats of these experiments are summarized in Table S1 (Supplementary information). (f) Survival curves of *hsf*-*1* mutants in the control or treated with 50 *μ*M naringin; naringin could not further extend the mean lifespan of *C*. *elegans*. Statistical details and repeats of these experiments were summarized in Table S1 (Supplementary information).

The body bending behavior of *C*. *elegans* declines with aging [39]. In order to test if naringin could delay the decline of body bending with aging, the body movement of nematodes was analyzed. Our results showed that although both treated and nontreated animals showed a tendency of movement slowing down with aging, the treatment of naringin significantly slowed the declining of body movement with aging (Figure 2(a), Table S2). In addition, the level of lipofuscin, an endogenous intestinal autofluorescent, accumulates during the aging of *C*. *elegans* [3, 40]. Our results showed that the fluorescence intensity of intestinal lipofuscin in worms treated with naringin was reduced by 46.8% and 15.1% on the second day and fifth day, respectively (Figure 2(b), Table S3), indicating that naringin treatment suppressed lipofuscin accumulation.

### 3.3. Naringin Promotes the Stress Resistance of *C*. *elegans*

There is a strong correlation between stress resistance and lifespan of *C*. *elegans*. Several studies have shown that the lifespan of *C*. *elegans* is affected by oxidative stress or high temperatures [41, 42]. In order to study whether naringin affects stress resistance of *C*. *elegans*, wild-type N2 worms were pretreated with naringin, followed by oxidative stress or heat stress treatment. Our results showed that pretreatment with naringin increased the survival rate of worms under oxidative stress by 18.1% (Figure 2(c), Table S4). The treatment of naringin increased the survival rate of worms under thermal stress by 31.2% (Figure 2(d), Table S5). In addition, we used *Pseudomonas aeruginosa* (PA14), a pathogenic bacterium, to test if naringin affected the resistance of worms to pathogenic stress. Our results showed that naringin could not extend the lifespan of nematodes fed by *Pseudomonas aeruginosa* (Figure 2(e), Table S1).

HSF-1 is a transcriptional regulator of stress-induced gene expression and protein-folding homeostasis [43]. Therefore, we tested the effect of naringin on the deletion mutant *hsf*-*1*(*sy441*)*I*. Our results showed that naringin could not extend the lifespan of *hsf*-*1*(*sy441*)*I* (Figure 2(f), Table S1).

### 3.4. Naringin Delays the Progression of Aging-Related Diseases in *C*. *elegans* Models of AD and PD

Misfolded proteins accumulate with aging and lead to chronic toxic stress for cells [27, 34], which causes a variety of aging-related neurodegenerative diseases, such as Parkinson's disease, Alzheimer's disease, and Huntington's disease. Since naringin could extend the lifespan of *C*. *elegans*, we are wondering if naringin could also ameliorate protein toxicity stress in nematodes and delay the progression of neurodegenerative diseases.

**Figure 3:**
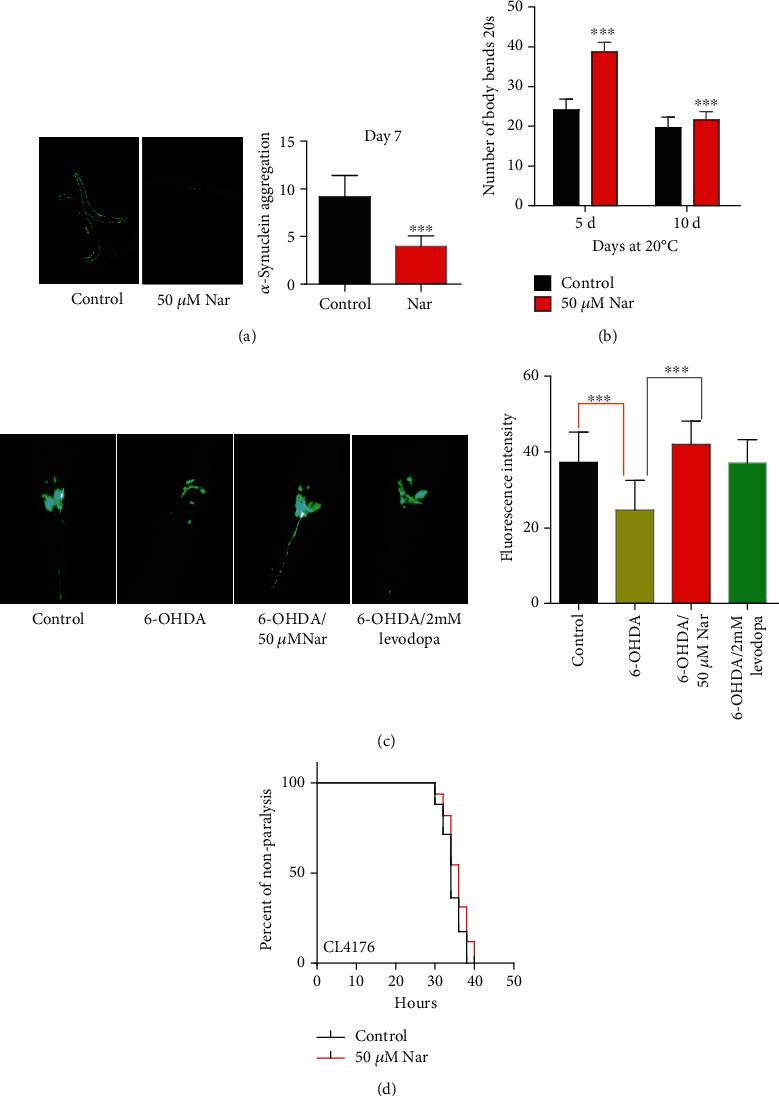
Naringin declines the progression of aging-related diseases. (a) Representative images of the *α*-synuclein in NL5901 treated with or without naringin. The aggregation of *α*-synuclein in NL5901 treated with or without naringin was captured with a Leica epifluorescence microscope and analyzed by using the image processing software ImageJ. 50 animals of each strain were scored in two independent trials. (b) Aging-related movements of NL5901 with nontreated control plates and 50 *μ*M naringin. The mean body movement speed is found in Table S2 (Supplementary information). (c) Representative images of the head neurons of BZ555 using different ways of rescue after 6-OHDA induction. After the rescue of 50 *μ*M naringin, the head neurons showed slow degenerative changes, and the degree of protection was basically consistent with levodopa which is the anti-Parkinson's disease drug. 40 animals of each strain were scored in each independent trial. Statistical details and repeats of these experiments are summarized in Table S6 (Supplementary information). (d) The paralysis phenotype associated with muscle A*β* expression is suppression by 50 *μ*M naringin treatment from the L3 stage larvae in the transgenic strain CL4176. Shown is the independent experiment with 70-100 animals in indicated time points after temperature upshift to 25°C. Statistical details and repeats of these experiments are summarized in Table S8 (Supplementary information).

Parkinson's disease is characterized by the accumulation of *α*-synuclein protein and degeneration of dopaminergic neurons in the substantia nigra [33, 34]. *C*. *elegans* does not have an *α*-synuclein homolog, so several transgenic *C*. *elegans* strains with human *α*-synuclein have been created to study the pathogenicity of *α*-synuclein. The transgenic strain NL5901 ([*unc*-*54p*::*α*-*synuclein*::*YFP+unc*-*119*(*+*)]) expresses *α*-synuclein protein fused with yellow fluorescent protein (YFP) [34] in the body wall muscle cells [33, 34]. We analyzed the aggregation of *α*-synuclein in worms treated with 50 *μ*M of naringin for 7 days. We found that naringin treatment significantly decreased the aggregation of *α*-synuclein (*p* < 0.001) (Figure 3(a), Table S6). In addition, the accumulation of *α*-synuclein aggregating in NL5901 is associated with locomotion and movement impairments [34]. Our results showed that naringin treatment could also increase the swimming bending behavior of *C*. *elegans* NL5901 (Figure 3(b), Table S2). Dopaminergic neurodegeneration can easily be induced by neurotoxins such as 6-hydroxydopamine (6-OHDA). Thus, another transgenic strain BZ555 (*P*^dat–1^::gfp) expressing green fluorescent protein (GFP) in dopaminergic neurons [35] was used to study the degeneration of dopaminergic neurons [33]. Fluorescent photographs of neurons in the head of the nematode BZ555 were taken after being exposed to 6-OHDA for 72 hours. Our results showed that BZ555 treated with 6-OHDA decreased the mean fluorescence intensity from 35.73 ± 2.129 to 18.94 ± 0.757 in the absence of naringin, whereas exposure to 50 *μ*M of naringin increased the mean fluorescence intensity to 38.82 ± 1.646 (Figure 3(c), Table S6). Our results also showed that BZ555 presents increased neurite blebbing under 6-OHDA treatment, while naringin treatment reduced half of the neurite blebbing caused by 6-OHDA. (Figure S1, Table S7). Above results suggest that naringin treatment slowed the degeneration of head neurons of *C*. *elegans* BZ555, with the degree of protection comparable to the anti-Parkinson's disease medicine levodopa (Figure 3(c), Table S6).

*β*-Amyloid is the main component of the extracellular plaques found in the brain of patients with Alzheimer's disease [27]. Temperature-induced expression of human beta-amyloid peptide (A*β*) in muscles of *C*. *elegans* CL4176 (*dvIs27* (*myo*-*3/A beta 1*-*42/let UTR*)*+pRF4* (*rol*-*6*(*su1006*)) leads to paralysis [27, 33]. Our results showed that 50 *μ*M of naringin could delay the temperature-induced paralysis of *C*. *elegans* CL4176 (Figure 3(d), Table S8), suggesting that naringin could suppress the toxicity of A-beta plaque.

### 3.5. The Effect of Naringin on the Lifespan Extension in *C*. *elegans* Depends on FOXO Homologous *daf*-*16*

**Figure 4:**
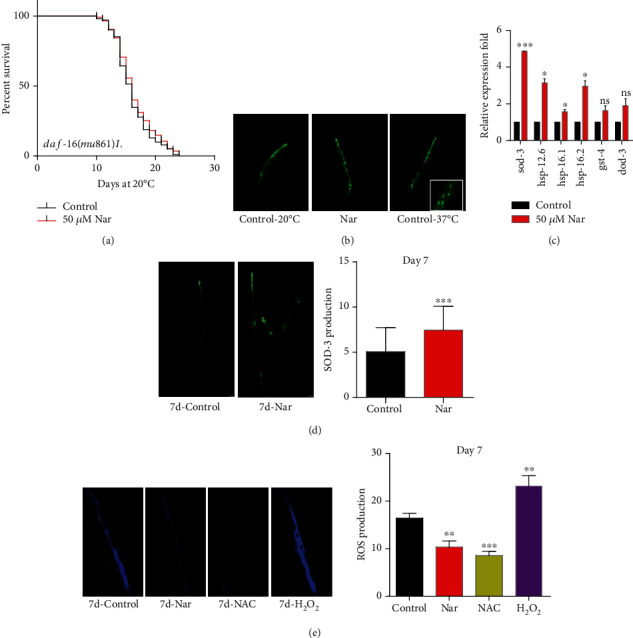
The effect of naringin on the lifespan extension in *C*. *elegans* depends on FOXO homologous *daf*-*16*. (a) Survival curves of *daf*-*16* mutants in the control or treated with 50 *μ*M naringin; naringin could not further extend the mean lifespan of *C*. *elegans*. (b) 50 *μ*M naringin could not lead to DAF-16 nuclear localization. DAF-16::GFP expressing worms were placed on the plates with 50 *μ*M naringin and control plates at 20°C for 48 h. (c) The mRNA level of genes downstream of *daf*-*16* in worms (N2) treated with or without 50 *μ*M naringin. The columns showed the mean value of two independent experiments with error bars representing SEM. ∗ represents *p* < 0.05, calculated using two-tailed *t*-test. Statistical details and repeats of these experiments are summarized in Table S9 (Supplementary information). (d) Representative images of the SOD-3::GFP report transgenic CF1553 with naringin or not; naringin enhanced the fluorescence intensity of SOD-3. The aggregation of SOD-3 in N2 treated with or without naringin was captured with a Leica epifluorescence microscope and analyzed by using the image processing software ImageJ. Statistical details and repeats of these experiments are summarized in Table S10 (Supplementary information). (e) Representative images of the degree of ROS with wild-type (N2) animals raised at 20°C on NGM plates via naringin, NAC (5 mM), and H_2_O_2_ (2 mM) treated; naringin impaired the fluorescence intensity of ROS. The aggregation of ROS in N2 treated with or without naringin was captured with a Leica epifluorescence microscope and analyzed by using the image processing software ImageJ. Statistical details and repeats of these experiments are summarized in Table S10 (Supplementary information).

In *C*. *elegans*, the transcription factor FOXO homologous *daf*-*16* plays a critical role in stress resistance, longevity, development, fat accumulation, and reproductivity [44, 45]. Our results showed that naringin could not extend the lifespan of *daf*-*16* null mutant *daf*-*16*(*mu86*)*I* (Figure 4(a), Table S1). Upon activation, *daf*-*16* transferred from the cytoplasm to the nucleus and activates the expression of downstream genes [46]. But our results showed that there was no significant increase in *daf*-*16* nuclear translocation in nematodes treated with naringin (Figure 4(b)). So we investigated if naringin could affect the mRNA level of *daf*-*16* regulated genes *sod*-*3*, *gst*-*4*, *dod*-*3*, *hsp12*.*6*, *hsp16*.*1*, and *hsp*-*16*.*2* [47, 48]. The mRNA expression level of genes *sod*-*3*, *hsp12*.*6*, *hsp16*.*1*, and *hsp*-*16*.*2* was significantly increased in wild-type N2 worms treated with naringin (*p* < 0.05). Among them, the mRNA expression of *sod*-*3* was increased up to 4-fold. No significant changes were found in the mRNA expression level of genes *gst*-*4* and *dod*-*3* in worms treated with naringin (*p* > 0.05) (Figure 4(c), Table S9). In addition, we monitored the fluorescence intensity of SOD-3::GFP in report transgenic strain CF1553; the naringin treatment significantly increased the GFP intensity (*p* < 0.001) (Figure 4(d), Table S10). SOD-3 is a mitochondrial superoxide dismutase which is involved in antioxidative stress [49]. Thus, we use the wild-type nematodes N2 to quantify the ROS in the body. We found that naringin treatment significantly lowered ROS (Figure 4(e), Table S10). These findings showed that naringin extended the lifespan of worms by activating *daf*-*16*.

**Figure 5:**
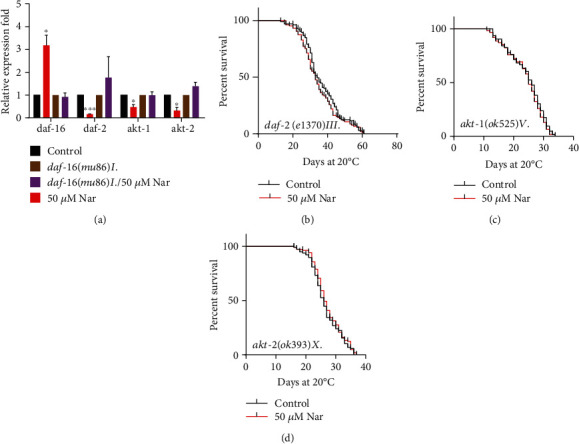
Naringin could affect the mRNA expression and not extend the lifespan of *daf*-*2*, *akt*-*1*, *and akt*-*2* mutants in the IIS pathway. (a) The mRNA expression level of genes associated with DAF-16 and itself. The columns showed the mean value of two independent experiments with error bars representing SEM. ∗ represents *p* < 0.05, calculated using two-tailed *t*-test. Statistical details and repeats of these experiments are summarized in Table S9 (Supplementary information). (b–d) Survival curves of *daf*-*2*, *akt*-*1*, *and akt*-*2* mutants in the control or treated with 50 *μ*M naringin; naringin could not further extend the mean lifespan or control plates without naringin. Statistical details of mutants and repeats of these experiments are summarized in Table S1 (Supplementary information).

In *C*. *elegans*, the insulin-like ligands interact with DAF-2/insulin receptors to activate the phosphoinositide 3-kinase age-1/PI3K, which regulates the activity of kinases AKT-1, AKT-2, and SGK-1 through phosphorylation of PDK-1. AKT regulates the activity of multiple downstream targets, including DAF-16/FOXO transcription factor [44]. Therefore, we detected the mRNA expression level of genes associated with DAF-16 and itself. The results showed that in naringin-treated groups, the relative expression levels of *daf*-*2*, *akt*-*1*, and *akt*-*2* were downregulated to 0.161 ± 0.014, 0.472 ± 0.108, and 0.317 ± 0.150, respectively. It was worth noticing that the relative expression level of *daf*-*16* was significantly upregulated to 3.184 ± 0.445. However, the naringin-treated group did not show significant differences in the expression of *daf*-*2*, *akt*-*1*, *akt*-*2*, as well as *daf*-*16* in *daf*-*16* null mutant *daf*-*16*(*mu86*)*I* (Figure 5(a), Table S9). So we studied the effect of naringin on long-lived mutant *daf*-*2*(*e1370*)*III* and loss of function mutants *akt*-*1*(*ok525*)*V* and *akt*-*2*(*ok393*)*X*. Our results showed that naringin could not prolong the lifespan of *daf*-*2*(*e1370*)*III* (Figure 5(b), Table S1), *akt*-*1*(*ok525*)*V*, and *akt*-*2*(*ok393*)*X* (Figures 5(c) and 5(d), Table S1).

### 3.6. Naringin Does Not Act on the Germline Signaling Pathway

**Figure 6:**
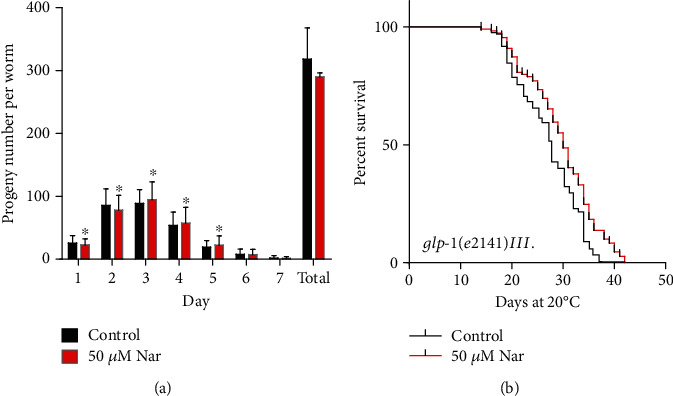
Naringin does not act on germline signaling. (a) The offspring of each animal was counted daily at each concentration, and total reproduction outputs were determined by each group. The error bar represent the standard deviation (SD), and no value reached the significance limit of *p* < 0.05 by independent *t*-test. Statistical details and repeats of these experiments are summarized in Table S11 (Supplementary information). (b) Survival curves of *glp*-*1* mutants in the control or treated with 50 *μ*M naringin; naringin could further extend the mean lifespan relative to control plates without naringin. Statistical details and repeats of these experiments are summarized in Table S1 (Supplementary information).

Reduced fertility and slower growth could extend the lifespan of *C*. *elegans* [50]. To test if naringin could affect the reproduction of *C*. *elegans*, we measured the oviposition of animals treated with naringin. We found that the number of eggs in the treated group was less than that in the untreated group on the 1st and 2nd days but was more than that in the untreated group on the next three days. The naringin treatment did not significantly change the total number of progenies throughout the spawning cycle (Figure 6(a), Table S11).

To investigate if naringin could extend the lifespan of *C*. *elegans* by affecting reproduction, we selected the germ-related *glp*-*1* mutant *glp*-*1*(*e2141*)*III* to determine if naringin acts on the reproductive signaling pathway. Our results showed that naringin treatment further extended the lifespan of the long-lived mutant (Figure 6(b), Table S1), indicating that naringin did not extend the lifespan of nematodes through the germline signaling pathway.

### 3.7. Naringin Could Not Extend the Lifespan of Long-Lived Mutants in the Nutrition-Sensing Pathway

**Figure 7:**
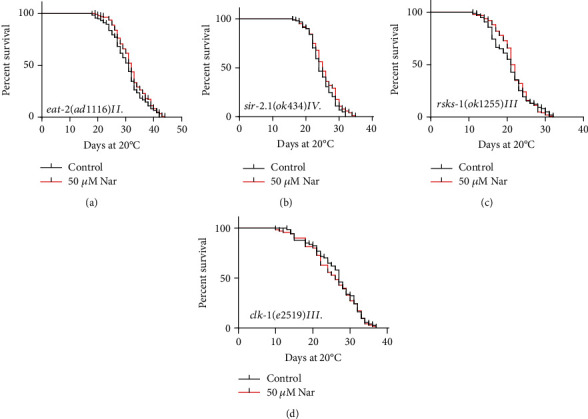
Naringin could not extend the lifespan of long-lived mutants in the nutrition-sensing pathway. (a–d) Survival curves of *eat*-*2*, *sir2*.*1*, *rsks*-*1*,and *clk*-*1* mutants in the control or treated with 50 *μ*M naringin; naringin could not further extend the mean lifespan of *C*. *elegans*. Statistical details and repeats of these experiments are summarized in Table S1 (Supplementary information).

Dietary restrictions (DR) reduce available nutrients, regulate metabolism, and can extend the lifespan of different organisms, including yeast and mammals [50]. To investigate if the role of naringin in extending the lifespan of *C*. *elegans* is related to DR, we measured the lifespan of *eat*-*2*(*ad1116*)*II*, a mutant with reduced consumption of food. Our results showed that naringin treatment could not significantly extend the lifespan of *eat*-*2*(*ad1116*)*II* (*p* = 0.182) (Figure 7(a), Table S1).

SIR-2.1 is a member of the Sir-2 family (NAD^+^-dependent protein deacetylases) and regulates nematode aging by interacting with various transcription factors including DAF-16. SIR2 protein has been associated with lifespan regulation in *C*. *elegans* [51]. We investigated whether naringin could act on SIR2 to extend the lifespan of *C*. *elegans* by using a null mutant strain *sir*-*2*.*1*(*ok434*)*IV*. Our results showed that naringin could not significantly extend the lifespan of *sir*-*2*.*1*(*ok434*)*IV* (*p* = 0.083) (Figure 7(b), Table S1).

The gene target of rapamycin (TOR) is a highly conserved nutrient-sensing kinase, which plays an important role in DR [52]. RSKS-1 is a homolog of TOR target nuclear subgroup S6 kinase (S6K) in nematodes. Here, our results show that naringin also could not further extend the lifespan of the loss-of-function mutant *rsks*-*1*(*ok1255*)*III* (*p* = 0.255) (Figure 7(c), Table S1).

In addition, mitochondrial involvement in DR-mediated life extension has been reported [52]. The gene *clk*-*1* encodes a mitochondrial hydroxylase necessary for the biosynthesis of ubiquinone. CLK-1 mutant *clk*-*1*(*e2519*)*III* is a long-lived mutant with mitochondrial respiratory dysfunction [53]. Our results showed that naringin did not significantly extend the lifespan of *clk*-*1*(*e2519*)*III* compared to the control group (Figure 7(d), Table S1).

## 4. Discussion

The natural product naringin has been reported to have multiple biological activities, such as antineoplastic, anti-inflammatory, and antioxidative activities [54]. So, we investigated if naringin could influence aging and neurodegenerative diseases. Our results showed that naringin treatment could increase the lifespan of *C*. *elegans*, delay the aging-related decline of body bending, relieve the accumulation of lipofuscin, enhance the stress resistance, and mitigate the aging-related diseases in models of PD and AD.

**Figure 8:**
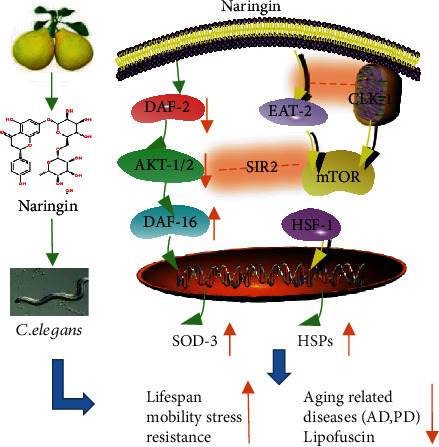
A dihydroflavonoid naringin increases stress tolerance, delays progression of aging-related diseases, and extends the lifespan of *C*. *elegans* via FOXO/DAF-16.

Naringin extends the lifespan of *C*. *elegans* cultured with both live and heat-killed bacteria, suggesting that naringin has biological activity in *C*. *elegans*. DAF-16 plays a crucial role in stress resistance, longevity, fat accumulation, and reproductive ability in *C*. *elegans* [47]. Naringin could not extend the lifespan of null mutated *daf*-*16*. Although no obvious nuclear translocalization was observed under treatment of naringin, naringin increased the mRNA expression of *daf*-*16* and the genes regulated by *daf*-*16*. Moreover, naringin inhibited the mRNA expression level of the genes upstream of *daf*-16 in the IIS pathway. Besides, naringin could not significantly extend the lifespan of long-lived mutants from genes upstream of *daf*-*16*, including *daf*-*2*, *akt*-*1*, and *akt*-*2*. These results suggest that naringin extends the lifespan of *C*. *elegans* by regulating the IIS pathway (Figure 8).

We also investigated if naringin could regulate other targets to extend the lifespan of *C*. *elegans*. It was found that naringin treatment further extended the lifespan of the long-lived *glp*-*1* mutant (Figure 6(b), Table S1), indicating that naringin did not extend the lifespan of nematodes through the germline signaling pathway.

Dietary restrictions (DR) that reduce available nutrients and regulate metabolism can extend the lifespan of different organisms, including yeast and mammals [50]. Nutrient-sensing pathways play a central role in aging and lifespan. Our results showed that naringin could not significantly extend the lifespan of long-lived mutants from genes in the nutrient-sensing pathway, such as *eat*-*2*, *sir*-*2*.*1*, *rsks*-*1*, and *clk*-*1* that modulate mitochondrial respiration. Above results suggest that either the effect of naringin on the lifespan extension is not strong enough to distinguish from the lifespan of the already long-lived mutants or these molecules are required for naringin to extend the lifespan of *C*. *elegans* (Figure 8). Another possibility is that at the molecular level, the mechanisms of DR appear embedded in the response to reduce energy availability, resulting in the emergence of an altered metabolic state that promotes health and longevity.

Here, we show that naringin could dramatically reduce the *α*-synuclein aggregation as well as alleviate paralysis in *C*. *elegans* models of AD and PD. Flavonoids chelate metal ions, preventing formation of free radicals and limiting the onset of PD [55]. Naringin is a kind of dihydroflavonoid. Our results show that naringin could increase the expression of SOD-3 and the scavenging activity of ROS. In addition, several studies showed the link between the IIS pathway and the nervous system. For instance, reduced IGF-1 signaling delays age-associated proteotoxicity in a mouse model of Alzheimer disease [56]. Similarly, attenuated IR substrate of IIS signaling in aging brains extended the lifespan in mice [57]. Likewise, basal IGF-1 activity has been reported to regulate ongoing neuronal activity in hippocampal circuits [58]. Interestingly, another study has shown that reduced IGF signaling mediated by FOXO could decrease the proteotoxic activities induced by A*β* hyperaggregation and oxidative stress [56]. Above reports together with our results indicate that naringin might mitigate the aging-related diseases through the IIS pathway.

## Data Availability

All the figures and tables used to support the findings of this study are included within the article and supplementary materials.

## Supplementary Materials

Supplementary Materials Figure S1: the effect of naringin on touch sensory neurons of PD. Table S1: the effect of naringin on lifespan. Table S2: the effect of naringin on body movement. Table S3: the effect of naringin on intestinal lipofuscin. Table S4: the effect of naringin on oxidative stress resistance. Table S5: the effect of naringin on heat resistance. Table S6: the effect of naringin on aging-related diseases. Table S7: the effect of naringin on touch sensory neurons of PD. Table S8: the effect of naringin on paralysis. Table S9: the effect of naringin on mRNA expression. Table S10: the effect of naringin on SOD-3 and ROS expression. Table S11: the effect of naringin on offspring. Table S12: list of primers used in gene expression assay.
