# Supplementary material for: A Dihydroflavonoid Naringin Extends the Lifespan of C. elegans and Delays the Progression of Aging-Related Diseases in PD/AD Models via DAF-16
Source: Oxid Med Cell Longev. 2020 Jul 31;2020:6069354. doi: 10.1155/2020/6069354 (PMC7422489; doi:10.1155/2020/6069354)
Supplement: Supplementary Materials — Figure S1: the effect of naringin on touch sensory neurons of PD. Table S1: the effect of naringin on lifespan. Table S2: the effect of naringin on body movement. Table S3: the effect of naringin on intestinal lipofuscin. Table S4: the effect of naringin on oxidative stress resistance. Table S5: the effect of naringin on heat resistance. Table S6: the effect of naringin on aging-related diseases. Table S7: the effect of naringin on touch sensory neurons of PD. Table S8: the effect of naringin on paralysis. Table S9: the effect of naringin on mRNA expression. Table S10: the effect of naringin on SOD-3 and ROS expression. Table S11: the effect of naringin on offspring. Table S12: list of primers used in gene expression assay. [file 6069354.f1.docx]

**A dihydroflavonoid naringin extends the lifespan of *C. elegans* and delays the progression of ageing-related diseases in PD/AD Models via DAF-16**

**Qing Zhu ^1^, Yuan Qu ^1^, Xiao-Gang Zhou ^1^, Jian-Ning Chen ^1^, Huai-Rong Luo ^1, 2, 3,*^, Gui-Sheng Wu ^1, 2, *^**

1 Key Laboratory for Aging and Regenerative Medicine, Department of Pharmacology School of Pharmacy, Southwest Medical University, Luzhou, Sichuan 646000, China.

2 Key Laboratory of Medical Electrophysiology, Ministry of Education, Institute of

Cardiovascular Research of Southwest Medical University, Luzhou, Sichuan 646000, China.

3 Central Nervous System Drug Key Laboratory of Sichuan Province, Luzhou, Sichuan 646000, China

* Corresponding author: Dr. Huai-Rong Luo, Dr. Gui-Sheng Wu;

Key Laboratory for Aging and Regenerative Medicine, Department of Pharmacology

School of Pharmacy, Southwest Medical University

319 Zhongshan Road, Luzhou, Sichuan 646000, China

Phone: +86 830-3160842; Fax: +86 830-3160842

E-mail address: [lhr@swmu.edu.cn](mailto:lhr@swmu.edu.cn), [wgs@swmu.edu.cn](mailto:wgs@swmu.edu.cn)

ORCID: <https://orcid.org/0000-0001-8912-6694>, <https://orcid.org/0000-0003-1913-9566>

**Supplementary information**

**Contents:**

**Figure S1. The effect of naringin on touch sensory neurons of PD**

**Table S1. The effect of naringin on lifespan**

**Table S2. The effect of naringin on body movement**

**Table S3. The effect of naringin on intestinal lipofuscin**

**Table S4. The effect of naringin on oxidative stress resistance**

**Table S5. The effect of naringin on heat resistance**

**Table S6. The effect of naringin on age-related diseases**

**Table S7. The effect of naringin on touch sensory neurons of PD**

**Table S8. The effect of naringin on paralysis**

**Table S9. The effect of naringin on mRNA expression**

**Table S10. The effect of naringin on SOD-3 and ROS expression**

**Table S11. The effect of naringin on offspring**

**Table S12. List of primers used in gene expression assay**

**Figure S1. The effect of naringin on touch sensory neurons of PD**

Figure S1. Naringin delays neuronal ageing. (A) Representative images of the head neurons of BZ555 using different ways of rescue after 6-OHDA induction. (B) Percent of animals showed branching/blebbing touch neurons in BZ555. After the rescue of 50 µM naringin, the head neurons showed increased non-branching rate. 40 animals of each strain were scored in each independent trail. Levodopa is the positive anti-Parkinson's disease drug. Values are mean percentage from three experiments, each experiment started with 40 worms. *P* values were calculated by two-tailed t-test. Statistical details were summarized in Table S7 (Supplementary information).

**Table S1. The effect of naringin on lifespan**

| **Figure** | **Strains** | **Treatments** | **Mean**  **Lifespan±SEM** | **P value**  **VS**  **control** | **（％）**  **Change in mean lifespan** | **N** |
| --- | --- | --- | --- | --- | --- | --- |
|  | **N2(WT)** |  |  |  |  |  |
| **1B** | EXP.1 | 20℃/Control | 19.072±0.490 |  |  | 83 |
|  | EXP.1 | 20℃/50 µM Naringin | 23.625±0.671 | *P*<0.001 | 23.873 | 64 |
|  | EXP.1 | 20℃/100 µM Naringin | 20.443±0.628 | 0.016 | 7.189 | 70 |
|  | EXP.1 | 20℃/200 µM Naringin | 21.595±0.638 | *P*<0.001 | 13.228 | 79 |
|  | EXP.1 | 20℃/500 µM Naringin | 18.667±0.518 | 0.857 | ＃ | 96 |
|  | EXP.2 | 20℃/Control | 19.879±0.665 |  |  | 58 |
| **1C** | EXP.2 | 20℃/25 µM Naringin | 22.185±0.619 | 0.002 | 11.600 | 81 |
|  | EXP.2 | 20℃/50 µM Naringin | 23.319±0.714 | *P*<0.001 | 17.304 | 69 |
|  | EXP.2 | 20℃/100 µM Naringin | 22.339±0.761 | 0.002 | 12.374 | 62 |
|  | EXP.2 | 20℃/200 µM Naringin | 21.228±0.623 | 0.050 | 6.786 | 79 |
|  | EXP.3 | 20℃/Control | 19.944±0.531 |  |  | 72 |
|  | EXP.3 | 20℃/25µM Naringin | 21.380±0.541 | 0.064 | ＃ | 71 |
|  | EXP.3 | 20℃/50µM Naringin | 23.87±0.686 | 0.019 | 19.685 | 69 |
|  | EXP.3 | 20℃/100µM Naringin | 21.098±0.580 | 0.032 | 5.786 | 82 |
|  | EXP.3 | 20℃/200µM Naringin | 20.733±0.544 | 0.109 | ＃ | 86 |
| **1D** | EXP.1 | 0µM Naringin/live bacteria | 15.438±0.394 |  |  | 64 |
|  | EXP.1 | 50 µM Naringin/live bacteria | 16.94±0.57 | 0.015 | 9.7 | 50 |
|  | EXP.1 | 0 µM Naringin/dead bacteria | 20.447±0.539 | *p*<0.001 |  | 85 |
|  | EXP.1 | 50 µM Naringin/dead bacteria | 22.731±0.655 | *p*<0.001 | 11.1 | 67 |
|  | EXP.2 | 0 µM Naringin/live bacteria | 15.84±0.385 |  |  | 75 |
|  | EXP.2 | 50 µM Naringin/live bacteria | 17.741±0.528 | 0.001 | 12 | 58 |
|  | EXP.2 | 0 µM Naringin /dead bacteria | 20.505±0.531 | *p*<0.001 |  | 91 |
|  | EXP.2 | 50 µM Naringin/dead bacteria | 23.5±0.608 | *p*<0.001 | 14.6 | 84 |
|  | EXP.3 | 0 µM Naringin /live bacteria | 15.039±0.338 |  |  | 77 |
|  | EXP.3 | 50 µM Naringin/live bacteria | 17.714±0.61 | *p*<0.001 | 17.7 | 49 |
|  | EXP.3 | 0 µM Naringin /dead bacteria | 20.179±0.54 | *p*<0.001 |  | 78 |
|  | EXP.3 | 50 µM Naringin/dead bacteria | 21.932±0.649 | *p*<0.001 | 8.6 | 74 |
| **2E** | EXP.1 | Control/PA14 | 11.862±0.159 |  |  | 188 |
|  | EXP.1 | 50 µM Naringin/PA14 | 11.17±0.123 |  | ＃ | 182 |
|  | EXP.2 | Control/PA14 | 11.917±0.173 |  |  | 133 |
|  | EXP.2 | 50 µM Naringin/PA14 | 11.254±0.149 |  | ＃ | 118 |
| **4A** | **CF1038/*daf-16(mu86)I.*** | | | | | |
|  | EXP.1 | 20℃/Control | 18.083±0.358 |  |  | 121 |
|  | EXP.1 | 20℃/50 µM Naringin | 18.274±0.349 | 0.764 | ＃ | 124 |
|  | EXP.2 | 20℃/Control | 16.639±0.409 |  |  | 97 |
|  | EXP.2 | 20℃/50 µM Naringin | 17.343±0.396 | 0.362 | ＃ | 88 |
|  | EXP.3 | 20℃/Control | 15.96±0.293 |  |  | 100 |
|  | EXP.3 | 20℃/50 µM Naringin | 16.18±0.283 | 0.721 | ＃ | 111 |
| **5A** | **CB1370/*daf-2(e1370) III.*** | | | | | |
|  | EXP.1 | 20℃/Control | 39.183±1.268 |  |  | 105 |
|  | EXP.1 | 20℃/50µM Naringin | 36.29±1.225 | 0.069 | ＃ | 107 |
|  | EXP.2 | 20℃/Control | 37.954±1.316 |  |  | 109 |
|  | EXP.2 | 20℃/50µM Naringin | 34.088±1.219 | 0.084 | ＃ | 113 |
|  | EXP.3 | 20℃/Control | 36.8±1.054 |  |  | 105 |
|  | EXP.3 | 20℃/50µM Naringin | 34.895±1.058 | 0.272 | ＃ | 105 |
| **5B** | **RB759/*akt-1(ok525)V.*** | | | | | |
|  | EXP.1 | 20℃/Control | 26.612±0.45 |  |  | 116 |
|  | EXP.1 | 20℃/50 µM Naringin | 27.276±0.462 | 0.392 | ＃ | 105 |
|  | EXP.2 | 20℃/Control | 25.517±0.388 |  |  | 116 |
|  | EXP.2 | 20℃/50µM Naringin | 26.350±0.440 | 0.071 | ＃ | 120 |
|  | EXP.3 | 20℃/Control | 27.197±0.492 |  |  | 117 |
|  | EXP.3 | 20℃/50 µM Naringin | 27.814±0.439 | 0.518 | ＃ | 129 |
| **5C** | **VC204/*akt-2 (ok393) X.*** | | | | | |
|  | EXP.1 | 20℃/Control | 20.738±0.749 |  |  |  |
|  | EXP.1 | 20℃/50 µM Naringin | 20.300±0.684 | 0.460 | ＃ | 65 |
|  | EXP.2 | 20℃/control | 24.508±0.763 |  |  | 70 |
|  | EXP.2 | 20℃/50µm Naringin | 24.032±0.759 | 0.468 | ＃ |  |
|  | EXP.3 | 20℃/Control | 25.538±0.788 |  |  | 65 |
|  | EXP.3 | 20℃/50 µM Naringin | 25.308±0.843 | 0.595 | ＃ | 52 |
| **2F** | **PS3551/*hsf-1(sy441)I.*** | | | | | |
|  | EXP.1 | 20℃/Control | 18.382±0.411 |  |  | 102 |
|  | EXP.1 | 20℃/50 µM Naringin | 17.721±0.424 | 0.346 | ＃ | 104 |
|  | EXP.2 | 20℃/Control | 18.458±0.407 |  |  | 107 |
|  | EXP.2 | 20℃/50 µM Naringin | 17.451±0.408 | 0.151 | ＃ | 113 |
|  | EXP.3 | 20℃/Control | 20.194±0.359 |  |  | 134 |
|  | EXP.3 | 20℃/50 µM Naringin | 20.481±0.307 | 0.766 | ＃ | 135 |
| **7A** | **DA1116/*eat-2(ad1116)Ⅱ.*** | | | | | |
|  | EXP.1 | 20℃/Control | 31.309±0.552 |  |  | 136 |
|  | EXP.1 | 20℃/50 µM Naringin | 32.889±0.468 | 0.182 | ＃ | 144 |
|  | EXP.2 | 20℃/control | 30.452±0.500 |  |  | 135 |
|  | EXP.2 | 20℃/50 µm Naringin | 31.923±0.454 | 0.084 | ＃ | 143 |
|  | EXP.3 | 20℃/control | 30.952±0.567 |  |  | 125 |
|  | EXP.3 | 20℃/50 µm Naringin | 32.015±0.502 | 0.287 | ＃ | 131 |
| **7B** | **VC199/*sir-2.1(ok434) IV.*** | | | | | |
|  | EXP.1 | 20℃/Control | 24.253±0.556 |  |  | 83 |
|  | EXP.1 | 20℃/50 µM Naringin | 24.978±0.546 | 0.304 | ＃ | 90 |
|  | EXP.2 | 20℃/Control | 25.289±0.420 |  |  | 121 |
|  | EXP.2 | 20℃/50 µM Naringin | 26.263±0.488 | 0.083 | ＃ | 99 |
|  | EXP.3 | 20℃/Control | 24.961±0.401 |  |  | 103 |
|  | EXP.3 | 20℃/50 µM Naringin | 25.894±0.399 | 0.033 | 3.7 | 123 |
|  | EXP.4 | 20℃/Control | 24.752±0.366 |  |  | 101 |
|  | EXP.4 | 20℃/50 µM Naringin | 25.485±0.408 | 0.085 | ＃ | 103 |
| **7C** | **RB1206/*rsks-1(ok1255) III.*** | | | | | |
|  | EXP.1 | 20℃/Control | 20.526±0.649 |  |  | 97 |
|  | EXP.1 | 20℃/50 µM Naringin | 19.135±0.528 | 0.255 | ＃ | 170 |
|  | EXP.2 | 20℃/Control | 20.796±0.505 |  |  | 98 |
|  | EXP.2 | 20℃/50 µM Naringin | 21.580±0.431 | 0.649 | ＃ | 100 |
|  | EXP.3 | 20℃/Control | 21.272±0.493 |  |  | 92 |
|  | EXP.3 | 20℃/50 µM Naringin | 20.879±0.443 | 0.408 | ＃ | 99 |
| **7D** | **CB4876/*clk-1(e2519) III.*** | | | | | |
|  | EXP.1 | 20℃/Control | 26.373±0.860 |  |  | 59 |
|  | EXP.1 | 20℃/50 µM Naringin | 26.667±0.739 | 0.874 | ＃ | 69 |
|  | EXP.2 | 20℃/Control | 26.257±0.734 |  |  | 74 |
|  | EXP.2 | 20℃/50 µM Naringin | 25.529±0.788 | 0.649 | ＃ | 70 |
|  | EXP.3 | 20℃/Control | 26.027±0.876 |  |  | 74 |
|  | EXP.3 | 20℃/50 µM Naringin | 26.330±0.785 | 0.914 | ＃ | 91 |
| **6B** | **CF1903/*glp-1(e2141) III.*** | | | | | |
|  | EXP.1 | 20℃/Control | 22.200±0.378 |  |  | 185 |
|  | EXP.1 | 20℃/50 µM Naringin | 23.606±0.426 | 0.006 | 6.3 | 142 |
|  | EXP.2 | 20℃/Control | 28.513±0.607 |  |  | 117 |
|  | EXP.2 | 20℃/50 µM Naringin | 30.636±0.677 | 0.005 | 7.4 | 107 |
|  | EXP.3 | 20℃/Control | 27.130±0.601 |  |  | 108 |
|  | EXP.3 | 20℃/50 µM Naringin | 29.624±0.648 | 0.001 | 9.2 | 109 |

The mean lifespan values were calculated by a log-rank (Kaplan-Meier) statistical test. *P* values were calculated for individual experiments, each consisting of control and experimental animals as the same time. N: total number of worms in each individual experiment. All statistical were calculated by using SPSS. “#”: no calculate ( *P>0.05*).

**Table S2. The effect of naringin on body movement**

| **Figure** | **Days** | **Strains** | **Treatments** | **Mean**  **Body movement±SEM** | **P value**  **VS**  **control** | **N** |
| --- | --- | --- | --- | --- | --- | --- |
| **2A** |  | **N2(WT)** |  |  |  |  |
|  | 5day | EXP.1 | 20℃/Control | 33.00±0.9375 |  | 20 |
|  |  | EXP.1 | 20℃/50 µM Naringin | 34.15±0.5861 | ＃ | 20 |
|  | 10day | EXP.1 | 20℃/Control | 24.15±1.122 |  | 20 |
|  |  | EXP.1 | 20℃/50 µM Naringin | 28.55±0.9417 | 0.005 | 20 |
|  | 5day | EXP.2 | 20℃/Control | 32.95±0.6133 |  | 20 |
|  |  | EXP.2 | 20℃/50 µM Naringin | 34.95±0.6427 | 0.030 | 20 |
|  | 10day | EXP.2 | 20℃/Control | 25.10±0.9400 |  | 20 |
|  |  | EXP.2 | 20℃/50 µM Naringin | 29.75±0.8882 | 0.001 | 20 |
| 3B |  | **NL5901** |  |  |  |  |
|  | 5day | EXP.1 | 20℃/Control | 24.120±0.381 |  | 50 |
|  |  | EXP.1 | 20℃/50 µM Naringin | 38.780±0.329 | *p*<0.001 | 50 |
|  | 10day | EXP.1 | 20℃/Control | 19.640±0.372 |  | 50 |
|  |  | EXP.1 | 20℃/50 µM Naringin | 21.680±0.285 | *p*<0.001 | 50 |
|  | 5day | EXP.2 | 20℃/Control | 24.480±0.214 |  | 50 |
|  |  | EXP.2 | 20℃/50 µM Naringin | 31.200±0.216 | *p*<0.001 | 50 |
|  | 10day | EXP.2 | 20℃/Control | 19.780±0.403 |  | 50 |
|  |  | EXP.2 | 20℃/50 µM Naringin | 22.780±0.222 | *p*<0.001 | 50 |

Body movement experiments were counted once every 20 seconds. *P* values were calculated by two-tailed t-test. N: total number of worms in each individual experiment. “#”: no calculate (because *P>*0.05).

**Table S3. The effect of naringin on intestinal lipofuscin**

| **Figure** | **Days** | **Strains** | **Treatments** | **Mean**  **Pigment±SEM** | **P value**  **VS**  **control** | **（％）**  **Change in mean lipofusion** | **N** |
| --- | --- | --- | --- | --- | --- | --- | --- |
| **2B** |  | **N2(WT)** |  |  |  |  |  |
|  | 2 day | EXP.1 | 20℃/Control | 4.127±0.100 |  |  | 50 |
|  |  | EXP.1 | 20℃/50µM Naringin | 2.608±0.098 | *P*<0.001 | 36.806 | 50 |
|  | 5 day | EXP.1 | 20℃/Control | 8.794±0.531 |  |  | 20 |
|  |  | EXP.1 | 20℃/50µM Naringin | 7.862±0.253 | 0.001 | 10.598 | 20 |
|  | 2 day | EXP.2 | 20℃/Control | 3.992±0.129 |  |  | 56 |
|  |  | EXP.2 | 20℃/50µM Naringin | 2.122±0.056 | *P*<0.001 | 46.844 | 59 |
|  | 5 day | EXP.2 | 20℃/Control | 9.648±0.477 |  |  | 20 |
|  |  | EXP.2 | 20℃/50µM Naringin | 8.193±0.195 | 0.007 | 15.081 | 20 |

The intestinal autofluorescence of lipofusion was analyzed on the 2th and 5th days of adult. In each experiment, the *P* values were calculated by two-tailed t-test and *P*<0.05.

**Table S4. The effect of naringin on oxidative stress resistance**

| **Figure** | **Strains** | **Treatments** | **Mean**  **Lifespan±SEM** | **P value**  **VS**  **control** | **（％）**  **Change in mean lifespan** | **N** |
| --- | --- | --- | --- | --- | --- | --- |
| **2C** | **N2(WT)** |  |  |  |  |  |
|  | EXP.1 | 20℃/Control | 10.356±0.137 |  |  | 73 |
|  | EXP.1 | 20℃/50 µM Naringin | 12.127±0.225 | *P*<0.001 | 17.101 | 55 |
|  | EXP.2 | 20℃/Control | 12.593±0.214 |  |  | 81 |
|  | EXP.2 | 20℃/50 µM Naringin | 14.873±0.414 | *P*<0.001 | 18.105 | 55 |

Oxidative stress resistance experiments were carried out with 20 mM of paraquat at 20℃. The mean lifespan and *P* values were calculated as described for Table S4. N: total number of worms in each individual experiment.

**Table S5. The effect of naringin on heat resistance**

| **Figure** | **Strains** | **Treatments** | **Mean**  **Lifespan±SEM** | **P value**  **VS**  **control** | **（％）**  **Change in mean lifespan** | **N** |
| --- | --- | --- | --- | --- | --- | --- |
| **2D** | **N2(WT)** |  |  |  |  |  |
|  | EXP.1 | 37℃/Control | 7.683±0.153 |  |  | 101 |
|  | EXP.1 | 37℃/50µM Naringin | 10.081±0.179 | *P*<0.001 | 31.212 | 99 |
|  | EXP.2 | 37℃/Control | 6.577±0.243 |  |  | 97 |
|  | EXP.2 | 37℃/50 µM Naringin | 11.105±0.18 | *P*<0.001 | 68.846 | 105 |

Heat resistance experiments were carried out at 35℃. The mean lifespan values were calculated by a log-rank (Kaplan-Meier) statistical test. *P* values were calculated for individual experiments, each consisting of control and experimental animals as the same time. N: total number of worms in each individual experiment.

**Table S6. The effect of** **naringin on age-related diseases**

| **Figure** | **Strains** | **Treatments** | **Mean** [**fluorescence**](C:/Users/Administrator/AppData/Local/youdao/dict/Application/8.5.1.0/resultui/html/index.html#/javascript:;) [**intensity**](C:/Users/Administrator/AppData/Local/youdao/dict/Application/8.5.1.0/resultui/html/index.html#/javascript:;)  **±SEM** | | **P value VS Control** | **N** |
| --- | --- | --- | --- | --- | --- | --- |
| **3A** | **NL5901** |  |  |  | |  |
|  | EXP.1 | 20℃/ Control | 9.161±0.318 |  | | 50 |
|  | EXP.1 | 20℃/ 50 µM Naringin | 3.973±0.155 | *p*<0.001 | | 50 |
|  | EXP.2 | 20℃/ Control | 8.999±0.505 |  | | 50 |
|  | EXP.2 | 20℃/ 50 µM Naringin | 3.375±0.113 | *p*<0.001 | | 50 |
| **3C** | **BZ555** |  |  |  | |  |
|  | EXP.1 | BZ555 | 35.73±2.129 |  | | 30 |
|  | EXP.1 | BZ555/6-OHDA | 18.94±0.757 | *p*<0.001 | | 30 |
|  | EXP.1 | BZ555/6-OHDA/ 50 µM Naringin | 38.82±1.646 | *p*<0.001 | | 38 |
|  | EXP.1 | BZ555/6-OHDA/2 mM Levodopa | 32.87±2.171 | *p*<0.001 | | 30 |
|  | EXP.2 | BZ555 | 37.28±1,254 |  | | 40 |
|  | EXP.2 | BZ555/6-OHDA | 24.67±1.249 | *p*<0.001 | | 40 |
|  | EXP.2 | BZ555/6-OHDA/ 50 µM Naringin | 42.03±0.969 | *p*<0.001 | | 40 |
|  | EXP.2 | BZ555/6-OHDA/ 2mM Levodopa | 37.16±0.967 | *p*<0.001 | | 40 |

The aggregation of α-synucleinin in NL5901 treated with or without naringin was captured with a Leica epifluorescence microscope and analyzed by using Image processing software Image J. Using different ways of rescue after 6-OHDA induction, quantity the fluorescent aggregation in each group. Levodopa is the positive anti-Parkinson's disease drug. *P* values were calculated by two-tailed t-test. N: total number of worms in each individual experiment. “#”: no calculate (because P>0.05).

**Table S7. The effect of naringin on touch sensory neurons of PD**

| **Figure** | **Treatments** | **Mean % of worms showing touch axon blebbing/branching**  **±SEM** | | **P value VS Control** |
| --- | --- | --- | --- | --- |
| **S1B** |  |  |  | |
|  | BZ555 | 34.17 ± 3.005 |  | |
|  | BZ555/6-OHDA | 85.83 ± 4.410 | *p*<0.001 | |
|  | BZ555/6-OHDA/ 50 µM Naringin | 45.00 ± 2.887 | 0.0015 | |
|  | BZ555/6-OHDA/2 mM Levodopa | 45.00 ± 3.819 | 0.0022 | |

Percent of animals showed branching/blebbing touch neurons in BZ555. After the rescue of 50 µM naringin, the head neurons showed increased non-branching rate. Levodopa is the positive anti-Parkinson's disease drug. *P* values were calculated by two-tailed t-test.

**Table S8. The effect of naringin on paralysis**

| **Figure** | **Strains** | **Treatments** | **Mean**  **Paralysis±SEM**  **(Hours)** | **P value**  **VS**  **control** | **（％）**  **Change in mean non-paralysis** | **N** |
| --- | --- | --- | --- | --- | --- | --- |
| **3D** | **CL4176** |  |  |  |  |  |
|  | EXP.1 | 25℃/Control | 34.103±0.282 |  |  | 78 |
|  | EXP.1 | 25℃/50µM Naringin | 35.025±0.264 | 0.028 | 2.703 | 80 |
|  | EXP.2 | 25℃/Control | 34.275±0.245 |  |  | 102 |
|  | EXP.2 | 25℃/50µM Naringin | 35.420±0.282 | 0.001 | 3.340 | 100 |

Stage L3 larva of strain CL4176 culturing at 15 °C were transferred to 25 °C and treated with 50 µM of naringin, and the paralysis was calculated every day. Shown is the independent experiment with 70-100 animals in indicated time points after temperature upshift. *P* values were calculated by a log-rank (Kaplan-Meier) statistical test. N: total number of worms in each individual experiment.

**Table S9. The effect of naringin on mRNA expression**

| **Figure** | **Strains** | **Gene** | **EXP.1** | **EXP.2** | **Mean±SEM** | ***P* value**  **VS**  **control** |
| --- | --- | --- | --- | --- | --- | --- |
| **4C** | **N2(WT)** | ***sod-3*** | 4.861 | 4.876 | 4.869±0.008 | *P*<0.001 |
|  |  | ***gst-4*** | 1.355 | 1.894 | 1.625±0.270 | 0.146 |
|  |  | ***dod-3*** | 1.537 | 2.274 | 1.906±0.369 | 0.133 |
|  |  | ***hsp-12.6*** | 2.923 | 3.367 | 3.145±0.222 | 0.011 |
|  |  | ***hsp-16.1*** | 1.447 | 1.683 | 1.565±0.118 | 0.041 |
|  |  | ***hsp-16.2*** | 2.6787 | 3.249 | 2.964±0.286 | 0.020 |
| **5A** | **N2(WT)** | ***daf-16*** | 3.629 | 2.739 | 3.184±0.445 | 0.039 |
|  |  | ***daf-2*** | 0.175 | 0.146 | 0.161±0.014 | *P*<0.001 |
|  |  | ***akt-1*** | 0.364 | 0.579 | 0.472±0.108 | 0.039 |
|  |  | ***akt-2*** | 0.167 | 0.467 | 0.317± 0.150 | 0.045 |
|  | **CF1038/*daf-16(mu86)I.*** | ***daf-16*** | 0.751 | 1.092 | 0.922±0.171 | # |
|  |  | ***daf-2*** | 2.688 | 0.839 | 1.764±0.925 | # |
|  |  | ***akt-1*** | 1.149 | 0.841 | 0.995± 0.154 | # |
|  |  | ***akt-2*** | 1.216 | 1.557 | 1.387±0.171 | # |

Every value of number representatives the expression level of genes and was carried out using 2^–△△CT^ method and normalized to the expression of gene *cdc-42*.

In each experiment, control and experimental worms were conducted in parallel and repeated in two independent trials. *P* values were calculated by two-tailed t-test.

**Table S10. The effect of naringin on SOD-3 and ROS expression**

| **Figure** | **Strains** | **Treatments** | **Mean** [**fluorescence**](C:/Users/Administrator/AppData/Local/youdao/dict/Application/8.5.1.0/resultui/html/index.html#/javascript:;) [**intensity**](C:/Users/Administrator/AppData/Local/youdao/dict/Application/8.5.1.0/resultui/html/index.html#/javascript:;)  **±SEM** | **P value VS Control** | **N** |
| --- | --- | --- | --- | --- | --- |
| **4D** | **SOD-3::GFP** |  |  |  |  |
|  | EXP.1 | 20℃/Control | 4.237±0.303 |  | 40 |
|  | EXP.1 | 20℃/50µM Naringin | 6.681±0.291 | *P*<0.001 | 40 |
|  | EXP.2 | 20℃/Control | 5.067±0.421 |  | 40 |
|  | EXP.2 | 20℃/50µM Naringin | 7.453±0.418 | *P*<0.001 | 40 |
| **4E** | **N2(WT)** | **ROS** |  |  | 20 |
|  | EXP.1 | 20℃/Control | 14.58 ± 1.060 |  | 20 |
|  | EXP.1 | 20℃/50µM Naringin | 7.155 ± 0.717 | *P*<0.001 | 20 |
|  | EXP.1 | 20℃/5mM NAC | 7.363 ± 0.5849 | *P*<0.001 | 20 |
|  | EXP.1 | 20℃/2mM H2O2 | 25.04 ± 2.310 | *P*<0.001 | 20 |
|  | EXP.2 | 20℃/Control | 15.62 ± 1.094 |  | 20 |
|  | EXP2 | 20℃/50µM Naringin | 9.336 ± 1.209 | *P*<0.001 | 20 |
|  | EXP.2 | 20℃/5mM NAC | 9.058 ± 0.8530 | *P*<0.001 | 20 |
|  | EXP.2 | 20℃/2mM H2O2 | 20.33 ± 0.9256 | 0.002 | 20 |
|  | EXP.3 | 20℃/Control | 16.41 ± 1.031 |  | 20 |
|  | EXP.3 | 20℃/50µM Naringin | 10.37 ± 1.243 | *P*<0.001 | 20 |
|  | EXP.3 | 20℃/5mM NAC | 8.544 ± 0.905 | *P*<0.001 | 20 |
|  | EXP.3 | 20℃/2mM H2O2 | 23.19 ± 2.120 | 0.003 | 20 |

SOD-3 and ROS expression experiments were carried out at 20°C. *P* values were calculated by two-tailed t-test. N: total number of worms in each individual experiment.

**Table S11. The effect of naringin on offspring**

| **Figure** | **Days** | **Strains** | **Treatments** | **Mean**  **offspring±SEM** | **P value**  **VS**  **control** | **N** |
| --- | --- | --- | --- | --- | --- | --- |
| **6A** |  | **N2(WT)** |  |  |  |  |
|  | 1 | EXP.1 | 20℃/Control | 26.000±2.192 |  | 30 |
|  |  | EXP.1 | 20℃/50µM Naringin | 23.000±1.772 | *p*<0.001 | 30 |
|  | 2 | EXP.1 | 20℃/Control | 85.667±4.314 |  | 30 |
|  |  | EXP.1 | 20℃/50µM Naringin | 78.033±1.589 | *p*<0.001 | 30 |
|  | 3 | EXP.1 | 20℃/Control | 88.833±3.997 |  | 30 |
|  |  | EXP.1 | 20℃/50µM Naringin | 94.733±5.210 | *p*<0.001 | 30 |
|  | 4 | EXP.1 | 20℃/Control | 53.633±4.532 |  | 30 |
|  |  | EXP.1 | 20℃/50µM Naringin | 57.567±1.277 | *p*<0.001 | 30 |
|  | 5 | EXP.1 | 20℃/Control | 19.167±1.896 |  | 30 |
|  |  | EXP.1 | 20℃/50µM Naringin | 22.533±2.658 | *p*<0.001 | 30 |
|  | 6 | EXP.1 | 20℃/Control | 7.933±1.496 |  | 30 |
|  |  | EXP.1 | 20℃/50µM Naringin | 7.700±1.514 | 0.550 | 30 |
|  | 7 | EXP.1 | 20℃/Control | 1.867±0.716 |  | 30 |
|  |  | EXP.1 | 20℃/50µM Naringin | 1.600±0.502 | 0.110 | 30 |
|  | 1 | EXP.2 | 20℃/Control | 11.600±1.320 |  | 25 |
|  |  | EXP.2 | 20℃/50µM Naringin | 13.080±1.666 | 0.001 | 25 |
|  | 2 | EXP.2 | 20℃/Control | 89.520±5.192 |  | 25 |
|  |  | EXP.2 | 20℃/50µM Naringin | 96.320±5.536 | *p*<0.001 | 25 |
|  | 3 | EXP.2 | 20℃/Control | 129.280±6.932 |  | 25 |
|  |  | EXP.2 | 20℃/50µM Naringin | 82.640±8.135 | *p*<0.001 | 25 |
|  | 4 | EXP.2 | 20℃/Control | 87.720±7.416 |  | 25 |
|  |  | EXP.2 | 20℃/50µM Naringin | 65.280±5.550 | *p*<0.001 | 25 |
|  | 5 | EXP.2 | 20℃/Control | 31.960±6.151 |  | 25 |
|  |  | EXP.2 | 20℃/50µM Naringin | 22.000±3.367 | *p*<0.001 | 25 |
|  | 6 | EXP.2 | 20℃/Control | 3.760±1.260 |  | 25 |
|  |  | EXP.2 | 20℃/50µM Naringin | 11.600±2.028 | *p*<0.001 | 25 |
|  | 7 | EXP.2 | 20℃/Control | 0.000±0.000 |  | 25 |
|  |  | EXP.2 | 20℃/50µM Naringin | 3.520±0.994 | *p*<0.001 | 25 |
|  | total | EXP.2 | 20℃/Control | 318.172±35.068 |  |  |
|  |  | EXP.2 | 20℃/50µM Naringin | 289.793±4.627 | 0.133 |  |

The offspring of each animal was counted daily in each group and total reproduction outputs were determined by each group. *P* values were calculated by two-tailed t-test. N: total number of worms in each individual experiment.

**Table S12. List of primers** **used in gene expression assay**

| **Gene** | **Type** | **Sequence** |
| --- | --- | --- |
| ***cdc-42*** | F | 5’- CTGCTGGACAGGAAGATTACG -3’ |
|  | R | 5’- CTCGGACATTCTCGAATGAAG -3’ |
| ***sod-3*** | F | 5’-AGCATCATGCCACCTACGTGA-3’ |
|  | R | 5’-CACCACCATTGAATTTCAGCG-3’ |
| ***dod-3*** | F | 5’-AAGCCATGTTCCCGAATGAG-3’ |
|  | R | 5’-GCTGCGAAAAGCAAGAAAATG-3’ |
| ***hsp-12.6*** | F | 5’-GTGATGGCTGACGAAGGAAC-3’ |
|  | R | 5’-GGGAGGAAGTTATGGGCTTC-3’ |
| ***hsp-16.1*** | F | 5’-GTCACTTTACCACTATTTCCGTCCAGCTCAACGTTC-3’ |
|  | R | 5’-CAACGGGCGCTTGCTGAATTGGAATAGATCTTCC-3’ |
| ***hsp-16.2*** | F | 5’-CTGCAGAATCTCTCCATCTGAGTC-3’ |
|  | R | 5’-AGATTCGAAGCAACTGCACC-3’ |
| ***gst-4*** | F | 5’- TCCGTCAATTCACTTCTTCCG -3’ |
| ***daf-16*** | R  F  R | 5’- AAGAAATCATCACGGGCTGG -3’  5’- ATCGTGTGCTCAGAATCC -3’  5’- ATGAATATGCTGCCCTCC -3’ |
| ***daf-2*** | F | 5’- CGGTGCGAAGAGAGGATATT-3’ |
|  | R | 5’-TACAGAGGTCGCCGTTACTG -3’ |
| ***akt-1*** | F | 5’-AGCCTAAGGAAGGACAAC -3’ |
|  | R | 5’-ATGAATCCAACGCTGACG -3’ |
| ***akt-2*** | F | 5’-ATTCAGCGAAGCACGAAC -3’ |
|  | R | 5’-CACAACCCAAAGTCAGTG -3’ |
